# Effects of the Supplementation of Lysophospholipids through Pelleted Total Mixed Rations on Blood Biochemical Parameters and Milk Production and Composition of Mid-Lactation Dairy Cows

**DOI:** 10.3390/ani10020215

**Published:** 2020-01-28

**Authors:** Yuhua He, Rongzhen Zhong, Long Cheng, Peihua You, Yiyong Li, Xuezhao Sun

**Affiliations:** 1 The Innovation Centre of Ruminant Precision Nutrition and Smart and Ecological Farming, Jilin Agricultural Science and Technology University, Jilin 132109, China; yhyttt0235@sina.com; 2 Jilin Inter-regional Cooperation Centre for the Scientific and Technological Innovation of Ruminant Precision Nutrition and Smart and Ecological Farming, Jilin 132109, China; zhongrongzhen@iga.ac.cn (R.Z.); ph-u@163.com (P.Y.); liyiyongfeed@163.com (Y.L.); 3 Northeast Institute of Geography and Agroecology, Chinese Academy of Sciences, Changchun 130102, China; 4 Faculty of Veterinary and Agricultural Sciences, Dookie Campus, The University of Melbourne, Victoria 3647, Australia; long.cheng@unimelb.edu.au; 5 Portal Agri-Industries Co., Ltd., Nanjing 211803, China

**Keywords:** lysophospholipids, pelleted total mixed ration, blood biochemical parameters, efficiency, cattle, feed additive, lactation

## Abstract

**Simple Summary:**

Dietary supplementation of lysophospholipids improves ruminant growth performance and may increase milk production in dairy cows. Pelleted total mixed rations are increasingly used in ruminant production systems. However, the effects of lysophospholipid supplementation in a pelleted total mixed ration for dairy cows have not been reported before. In this study, we fed dairy cows pelleted total mixed rations containing 0 or 0.5 g of lysophospholipids in a kilogram of diet and found that lysophospholipids did not increase milk and nutrient yields or improve milk quality, although the feed additive altered certain plasma biochemical parameters, which may be beneficial for animal health. We do not recommend lysophospholipids to be used as a feed additive in pelleted total mixed rations for dairy cows based on the current evidence we collected from this study.

**Abstract:**

Lysophospholipids (LPL), a new feed additive, were supplemented to a pelleted total mixed ration (TMR) of dairy cows to examine its effects on feed intake, production, and composition of milk and plasma biochemical parameters. Two dietary treatments included diets supplemented without (control diet; CON) or with LPL at a dose of 0.5 g/kg of pelleted TMR. Twelve multiparous, mid-lactation, Holstein cows (Bodyweight 730 ± 9.3 kg; 100 ± 6.0 days in milk) were randomly assigned to one of the two dietary treatments with a 42-day measurement period after a 14-day adaptation period. Feed and water were provided *ad libitum*. Feed intake and milk yields were recorded daily, blood samples were collected fortnightly, and milk samples weekly. The results showed that the supplementation of LPL did not change feed dry matter intake, milk yields, and milk composition. However, it increased total protein and globulin and the activity of alkaline phosphatase and decreased total cholesterol in plasma. This study suggests that LPL may have beneficent effects in animal health but might be not a feasible feed additive to increase production for dairy cows fed a pelleted TMR.

## 1. Introduction

Dairy cattle production is important worldwide for consumers and dairy farmers. In 2017 the world milk production was as high as 811 million tonnes [1]. With the increase in living standards and the growth of world population, the demand for milk is increasingly driving animal scientists to reduce the water footprint in dairy production [2] and to seek approaches to produce more milk and also improve its quality.

Lysophospholipids (LPL) including lysophosphatidylcholine, lysophosphatidic acid, lysophosphatidylethanolamine, and lysophosphatidylinositol, are made from phospholipids with a fatty acid chain cleaved during enzymatic hydrolysis [3]. This group of compounds have been widely used in non-ruminant feed to improve productive performance [4,5,6,7]. Recently, several studies have attempted to apply LPL to ruminants, including sheep [8], beef cattle [9] and dairy cows [10,11]. The responses of animal performance to the dietary supplementation of LPL are inconsistent in sheep and beef cattle [8,9], but milk protein and milk yields were improved and LPL is recommended to be a potential feed additive for dairy cows [11].

The feeding of dairy cows has shifted in the past half a century from concentrate and forage in a diet fed separately to a total mixed ration (TMR) due to a range of advantages over the conventional feeding [12]. A TMR aims to ideally allow cows to consume a complete diet with balanced nutrients, and thus stabilize rumen environment with less fluctuation in ruminal pH value, ammonia concentration, and ruminal fermentation to achieve a high yield, no or little loss of live weight during the lactation period and a low incidence rate of rumen acidosis. However, dairy cows can preferentially select concentrate components over large-sized forage components in a TMR, leading to bulky feed left in the trough [13,14]. This behavior results in imbalanced nutrients in ingested feed, which can be prevented with feed pelleting. Pelleted TMR has been increasingly used in the production systems for housed ruminants such as sheep [8,15,16], goats [17,18], beef cattle [19], with improved animal performance [20]. Recently, attempts have been made to use pelleted TMR in dairy cows [21].

The study on the dietary supplementation of LPL by Lee et al. [11] was conducted in lactating cows fed with TMR. How cows respond to the supplementation of LPL to pelleted TMR is unknown. During the process of feed pelleting, feed ingredients are subject to various treatments, such as steaming, conditioning, and pressing, which might affect the biological activity of LPL. However, non-ruminants still positively respond to LPL when supplemented in a pelleted diet [22]. Therefore, the objective of this study was to investigate the production and composition of milk and blood biochemical parameters in mid-lactation dairy cows with LPL supplemented through a pelleted TMR.

## 2. Materials and Methods

Animal ethics approval for this study was granted in advance by the Animal Care and Ethics Committee of Jilin Agricultural Science and Technology University (Approval No. 2018002).

### 2.1. Animals and Experiment Design

Twelve multiparous Holstein cows with similar body weight (mean ± SD; 730 ± 9.3 kg) and days in milk (100 ± 6.0) were selected for the experiment and randomly assigned to one of the two dietary treatments, with six cows in each group. The two dietary treatments included (1) control diet (CON) and (2) control diet supplemented with 0.5 g/kg of LPL. A randomized design was employed for the comparison of the two treatments (CON *versus* LPL) in the study. The experiment lasted for 56 days with the first 14 days for animals to adapt to their designated pelleted TMR and then 42 days for measurements.

### 2.2. Feed, Feeding and Animal Management

The diets were formulated according to NRC nutrient requirements for mid-lactation dairy cows [23]. The ingredients and chemical composition of the diets are listed in Table 1.

Flint corn grains were ground to pass a 5.0 mm screen and other ingredients to pass a 4.0 mm screen. Corn germ meal was the residue after solvent extraction for the lipid of corn germs from wet grinding of corn kernels. Sunflower seed meal was the lipid extraction residue of sunflower seeds with a ratio of shell to kernel roughly at 35:65. All ingredients were mixed and pelleted with steam conditioning at a 75 °C exit temperature using a pelleting machine (model YPM508E, Jiangsu Yongli Machinery Co., Ltd., Liyang, China). The diameter of the pellets was 4.2 mm. Feed pellets were manufactured in one batch at the Chifeng Subsidiary Company of Jiangsu Portal Agri-Industries Co., Ltd. (Chifeng, China).

During the experiment, cows were housed in a free stall barn with rubber mat bedding and the exercise areas had a concrete floor and a scraper system for manure removal. During feeding, the cows were individually held in pens (3.0 m × 5.0 m) for 1.5–2 h each feeding and fed twice a day at 8:00 and 20:00 and feed allowance was set to allow around 5% refused. Water was supplied *ad libitum*. Cows were milked three times a day at 7:00, 16:00 and 24:00 using an automatic milk machine mounted with a milk recording cup (model HP101 milking machine, Alfa Laval Agricultural Corp., Guangzhou, China). 

### 2.3. Sampling and Sample Analysis

Feed allowance and refusal were quantified daily for each cow to calculate feed intake. Feed dry matter (DM) content was determined by drying at 105 °C until a constant weight was reached. For the analysis of chemical composition, feed samples were dried at 65 °C for 48 h, ground to pass through a 1-mm screen (Wiley mill, Arthur H. Thomas, Philadelphia, PA, USA) and stored in a fridge. They were analyzed for CP, lipid, starch, Ca and total P [24,25], and for NDF and ADF [26] using heat-stable α-amylase and sodium sulfite and expressed exclusive of residual ash.

Milk production volume was recorded daily for each cow and averaged weekly and a 100 mL milk sample was collected weekly at each milking from each cow. Morning and afternoon milk samples were stored at 4 °C and pooled with evening samples over each cow. Milk compositions were analyzed immediately using an automatic milk analyzer with mid-infrared wave-band (MILKYWAY-2; Lohand Biological Science Ltd., Hangzhou, Zhejiang, China). The energy-corrected milk (ECM) was calculated as follows: ECM (kg) = 0.3246 × milk yield (kg) + 13.86 × milk fat yield (kg) + 7.04 × milk protein yield (kg) [27].

Blood samples (5 mL) were collected into Li-heparin treated tubes from coccygeal vessels from all experimental cows before morning milking on days 0, 14, 28 and 42 of the measurement period, kept over ice and transferred to the laboratory within 1 h, and then centrifuged at 2500× *g* for 15 min at 4 °C. Plasma was stored at −20 °C and thawed overnight at 4 °C for the analysis of biochemical parameters, including total protein (TP), albumin (ALB), globulin (GLOB), glucose (GLU), plasma urea nitrogen (PUN), triglyceride (TG), total cholesterol (TC) concentrations and alkaline phosphatase (ALP) activity. The analysis was performed using a commercial blood analyzer (Hitachi 7020, Beijing, China) and commercial kits (Jiancheng Biology Co., Nanjing, China).

### 2.4. Statistical Analyses

Data for blood parameters and the production and composition of milk were analyzed using GenStat 19th edition (VSN International, Hemel Hempstead, UK, 2017) [28] in the REML model for repeated measurements being set with equally spaced time points and split-plot in time with repeated measures. The model is as follows:

Y_ijk_ = μ +T_i_ + S_j_ × (1 + T_i_) + A_k_ + Ɛ_ijk_

where μ is the overall mean; T_i_ is the fixed effect of treatment (CON *versus* LPL); S_j_ is the fixed effect of sampling time (week 0, 1, 2, 3, 4, 5, 6 and 7 for milk data and day 0, 14, 28 and 42 for blood data); A_k_ is the random effect of animals (k = 6), A_k_ ~ N(0, σ_A_^2^I) and Ɛ_ijk_ is the random error term, Ɛ_ijk_ ~ N(0, σ_Ɛ_^2^I). Multiple comparison was made in the Duncan’s test and differences were considered significant if *p* ≤ 0.05.

## 3. Results

### 3.1. Feed Intake, Milk Production, and Compositions

The supplementation of LPL did not increase feed dry matter intake (DMI; CON 25.2 kg/day *versus* LPL 26.2 kg/day, *p* = 0.174).

There were significant interactions (*p* < 0.05) between treatment and sampling time for milk yield, ECM and yields of milk fat, proteins, solids not fat (SNF), lactose and ash (Table 2). These indexes were not significantly (*p* > 0.05) affected by the supplementation of LPL, but they changed with sampling time (*p* < 0.001). For example, milk yield decreased with sampling time and the extent of decrease tended to be greater for the LPL treatment than the control (27% *versus* 12%, *p* = 0.061). The tendency was similar for the other indexes. 

In terms of nutrient contents in milk, treatment by sampling time interaction and treatment did not have significant effects (*p* > 0.05). Milk fat content increased with sampling time (*p* < 0.001) and the contents of milk protein, SNF, lactose and ash did not significantly change with time (*p* > 0.180). 

Feed conversion efficiency (FCE; milk yield/DMI and ECM/DMI) and nitrogen use efficiency (milk N/feed intake N; NUE) were significantly affected by the interaction between sampling time and the supplementation of LPL (*p* < 0.001). In weeks 0 and 1 the cows supplemented with LPL had numerically higher FCE and NUE than the cows in the CON group (*p* > 0.05). In week 2 the two groups had almost the same values of FCE and NUE. From week 3, FCE and NUE were lower for the LPL group than the CON group and *p* values reached the significance level of 0.05 from week 4.

### 3.2. Blood Biochemical Parameters

Significant interactions between treatment and sampling time were not observed for the eight plasma biochemical parameters assayed. The supplementation of LPL significantly increased the concentrations of TP and GLOB in plasma, by 11% (*p* = 0.017) and 19% (*p* = 0.004), respectively. It increased the activity of ALP by 39% (*p* < 0.001) but decreased the concentration of TC by 15% (*p* = 0.027; Table 3). Other plasma biochemical parameters including ALB, TG, PUN and GLU were not significantly affected (*p* > 0.252) by the supplementation of LPL. The concentrations of total protein tended to fluctuate with time (*p* = 0.097). The concentrations of PUN and GLU significantly increased with time (*p* < 0.001).

## 4. Discussion

To the best of our knowledge, there are only two studies [10,11] in the literature investigating the effects of dietary LPL supplementation in dairy cows. The findings in this study are compared mainly to these two studies and some other studies on other ruminants and non-ruminants.

The lack of difference in feed intake between CON and LPL in this study is consistent with the reports by Rico et al. [10] who supplemented LPL at 10 g/day (equivalent to about 0.35 g/kg of dietary DM) to a TMR for lactating cows and by Lee et al. [11] who supplemented LPL at 0.5 or 0.75 g/kg of dietary DM, suggesting that any improvements in animal performance by LPL, if existent, would come from increased utilization efficiency of feed ingested rather than more nutrients supplied. In this study, we did not find improved FCE and NUE, indicating that LPL supplementation increased neither feed intake nor feed utilization.

Our study did not find a significant increase in milk yield, ECM and milk nutrient yields. As there were only six cows in each treatment group in the current study, this is a preliminary investigation and the power to detect treatment effects is limited. Nevertheless, this finding is consistent with the study by Rico et al. [10] who failed to find an increase in milk yield with the supplementation of LPL in dairy cows, but contrasts with the finding by Lee et al. [11]. In the study by Rico et al. [10], a crossover design was adopted with a 10-day washout phase and a 10-day measurement phase in each experimental period. The measurements were taken 5 and 10 days after the supplementation of LPL. The study by Lee et al. [11] was conducted in a 4 x 4 Latin square design with a 14-day adaptation period followed by a 7-day measurement period. The effects of LPL were observed between 1 and 7 days after the supplementation. In our study, the measurements were made between 15 and 56 days after the start of LPL supplementation. Milk yield was numerically higher for the LPL treatment than the control at the beginning of the measurement period and decreased with time at a greater rate for the LPL treatment than for the control, resulting in lower milk yield at the late phase of the measurements. As a result, overall, LPL supplementation did not have a significant effect in our study. Our results may be more reliable than the previous studies [10,11] as we investigated long-term effects rather than short-term effects as these previous studies did. It is worthwhile to note that our study and the studies by Rico et al. [10] and Lee et al. [11] all had a limited number of experimental animals. Experiments with a large scale are needed in future studies.

In addition to the duration of experiments, other factors, such as the source, method of manufacturing, dose, degradation in the rumen and rumen bypass of LPL, as suggested by Lee et al. [11], are necessary considerations for the comparison of results from different studies. The production responses to LPL are inconsistent in other ruminant species, such as beef [9] and sheep [8], whereas they are relatively consistent in non-ruminants [6,29,30], suggesting the complexity of LPL with ruminants. Thus, more studies are needed for a solid conclusion.

In our study, nutrient contents were unaffected by the supplementation of LPL, which is consistent with the study by Lee et al. [11] and with the study by Rico et al. [10], except fat content. Rico et al. [10] found that the supplementation of LPL to a diet with no added oil increased milk fat content but the fat content decreased progressively when the diet was changed to 20 g of oil added to 1 kg of diet. We added 7 g of calcium soap of long-chain fatty acids to 1 kg of diet and no effects were observed. The discrepancy among these studies is difficult to explain. In a sheep study by Huo et al. [8], lipase in serum was decreased with the supplementation of LPL. The dietary supplementation of LPL might affect animal lipid metabolism.

In dairy cows, metabolic status is normally evaluated with blood biochemical parameters [31]. Globulins are proteins in blood eliminating pathological microbes from the body and indicate the immune system status of the body. The increase of GLOB with the supplementation of LPL in this study may reflect the improvement of body in immunology. Total protein is the sum of GLOB and ALB. The increase of TP in plasma may just result from the increase of GLOB with LPL supplementation. Albumin is an indicator of nutrition status in the body and PUN, a waste product of protein metabolism in the liver, can be used to evaluate nitrogen excretion and utilization efficiency in cattle [32]. Albumin and PUN were unaffected in this study, suggesting the protein status of the body is not improved with LPL. On the other hand, GLU concentration in plasma was not changed with LPL, suggesting the energy status is not improved either.

Total cholesterol and TG are lipid metabolism-related blood biochemical parameters. The decrease in total cholesterol with the supplementation of LPL in this study suggests the lipid metabolism in blood was altered, which is in line with Huo et al. [8] who found a change of lipase activity in serum when LPL was supplemented to a sheep diet.

The activity of alkaline phosphatase (ALP) in blood indicates liver function for postpartum dairy cows [33] and is also associated with the deposition of calcium and phosphorus in the bone of animals [34]. In this study, LPL supplementation largely increased the activity of ALP in plasma, suggesting the liver function of lactating cows was enhanced and the loss of calcium and phosphorus from bones during lactation might be slowed down.

The concentration and structure of LPL before and after pelleting of the TMR were not monitored due to the lack of the facility in our laboratory. Such an analysis would provide direct evidence on the stability of LPL during pelleting. However, significant effects of LPL supplementation on blood parameters observed in this study and in the study by Huo et al. [8] indicate the presence of the biological activity of LPL in the TMR pellets, suggesting that the heat and humidity of pelleting did not, or at least not totally, inactivate LPL activity. This is consistent with the observations in non-ruminants with LPL supplemented to a diet for pelleting [22]. We would suggest that LPL is relatively stable during pelleting.

In this study, all ingredients were ground to pass a 4.0 mm screen, except corn grains which were ground to pass a 5.0 mm screen. It was suggested that the particle size in the diet for lactating cows should be over 31.2% physically effective NDF (peNDF) inclusive particles > 1.18 mm or 18.5% peNDF inclusive particles > 8 mm [35]. Apparently, the diet in this study had little peNDF and certainly could not meet the requirement of cows for peNDF. The lack of peNDF in a diet may result in acute or subacute ruminal acidosis. This issue was also raised in another study with pelleted TMR fed to lactating cows [21]. There were seven cows originally assigned to each of the two treatments in this study, and one cow in each treatment group became sick during the experiment and was taken out of the study. The sick cows had clinical signs of acidosis, although all other cows were normal in behaviour. This study was designed to investigate the effect of LPL only, but in future studies with pelleted TMR, acidosis and relevant indicators and parameters should be monitored. In practice, roughages might be supplied together with pelleted TMR to minimize the risk of acute and subacute rumen acidosis.

## 5. Conclusions

Lysophospholipids supplemented at a rate of 0.5 g/kg through a pelleted total mixed ration to mid-lactation cows did not alter milk yield and milk composition, but it altered certain plasma biochemical parameters. Further studies are warranted to draw a solid conclusion on the responses of dairy cows to lysophospholipids for future use as a feed additive.

## Figures and Tables

**Table 1:** Ingredients and nutrient contents of experimental diets supplemented without control diet (CON) or with lysophospholipids (LPL).

Item	Treatment	
	CON	LPL
Ingredient (g/kg of diet DM)		
Ground corn grains	244	244
Soybean meal	30	30
Corn germ meal	399	399
Sunflower seed meal	180	180
Sunflower seed shell	95	95
Ca-LCFA ^1^	11	11
Sodium bicarbonate	6	6
Premix (Mineral salts and vitamins) ^2^	35	35
Lysophospholipids		0.5
Nutrient contents ^3^ (g/kg of DM)		
Dry matter (DM) (g/kg of fresh weight)	893	905
Crude protein (CP)	166	163
Neutral detergent fiber (NDF)	311	308
Acid detergent fiber (ADF)	163	165
Lipid	35	34
Ash	65	64
Ca	6.7	6.8
Total P	4.7	4.8
NE_L_^4^ (Mcal/kg of DM)	1.44	1.42

^1^ Ca-LCFA, calcium soap of long-chain fatty acids; ^2^ Premix per kg contained 460 g of dried corn distillers grains with solubles, 360 g of limestone (38% Ca), 8.5 g of magnesium oxide (54% Mg), 32 g of P, 10 g of Fe, 3 g of Cu, 0.7 g of Mn, 1.3 g of Zn, 13 mg of Co, 33 mg of I, 600 mg of Se, 170,000 IU of vitamin A, 330,000 IU of vitamin D and 1100 IU of vitamin E; ^3^ The nutrient contents were measured values; ^4^ NE_L_, net energy for lactation, was estimated using NRC (2001) [23].

**Table 2:** Milk yield and compositions, feed conversion efficiency and nitrogen use efficiency of dairy cows fed experimental diets supplemented without (CON) or with (LPL) lysophospholipids.

Index	Treatment		SEM ^1^	Sampling Time							SEM	*p* Value		
	CON	LPL		Week 0	Week 1	Week 2	Week 3	Week 4	Week 5	Week 6		Treat	Time	Treat × Time
Milk yield (kg/d)	34.74	34.14	0.739	39.09 ^a 2^	37.58 ^a^	37.91 ^a^	32.45 ^b^	32.43 ^b^	30.43 ^b^	31.16 ^b^	0.946	0.579	<0.001	0.048
ECM ^3^ (kg/d)	35.76	34.81	0.722	38.76 ^a^	38.46 ^a^	38.52 ^a^	33.44 ^b^	33.56 ^b^	31.76 ^b^	32.50 ^b^	0.981	0.373	<0.001	0.008
Milk fat (%)	3.46	3.39	0.032	3.18 ^c^	3.40 ^b^	3.37 ^b^	3.46 ^ab^	3.45 ^ab^	3.53 ^ab^	3.56 ^a^	0.061	0.133	<0.001	0.208
Milk fat (kg/d)	1.20	1.15	0.025	1.24 ^a^	1.28 ^a^	1.28 ^a^	1.12 ^b^	1.12 ^b^	1.08 ^b^	1.11 ^b^	0.037	0.267	<0.001	0.006
Milk protein (%)	3.24	3.22	0.059	3.21	3.22	3.20	3.21	3.31	3.27	3.19	0.064	0.840	0.689	0.601
Milk protein (kg/d)	1.12	1.10	0.026	1.26 ^a^	1.21 ^a^	1.21 ^a^	1.04 ^b^	1.07 ^b^	0.99 ^b^	0.99 ^b^	0.034	0.512	<0.001	0.016
Milk SNF ^4^ (%)	8.70	8.75	0.156	8.40 ^b^	8.82 ^a^	8.59 ^ab^	8.79 ^a^	8.91 ^a^	8.86 ^a^	8.72 ^ab^	0.158	0.841	0.063	0.413
Milk SNF (kg/d)	3.02	2.98	0.070	3.28 ^a^	3.32 ^a^	3.26 ^a^	2.85 ^b^	2.89 ^b^	2.69 ^b^	2.71 ^b^	0.089	0.713	<0.001	0.018
Milk lactose (%)	4.79	4.81	0.083	4.70	4.84	4.67	4.82	4.89	4.90	4.78	0.087	0.847	0.180	0.302
Milk lactose (kg/d)	1.66	1.64	0.037	1.84 ^a^	1.82 ^a^	1.77 ^a^	1.56 ^b^	1.58 ^b^	1.49 ^b^	1.49 ^b^	0.049	0.699	<0.001	0.014
Milk ash (%)	0.70	0.71	0.012	0.70	0.72	0.69	0.71	0.72	0.72	0.71	0.014	0.526	0.389	0.327
Milk ash (kg/d)	0.24	0.24	0.006	0.27 ^a^	0.27 ^a^	0.26 ^a^	0.23 ^b^	0.23 ^b^	0.22 ^b^	0.22 ^b^	0.011	0.988	<0.001	0.005
Milk yield/DMI ^5^ (kg/kg)	1.34	1.28	0.028	1.58 ^a^	1.31 ^c^	1.42 ^b^	1.22 ^d^	1.23 ^cd^	1.26 ^cd^	1.14 ^d^	0.036	0.161	<0.001	<0.001
ECM/DMI (kg/kg)	1.38	1.30	0.027	1.57 ^a^	1.34 ^c^	1.44 ^b^	1.25 ^cd^	1.28 ^cd^	1.32 ^c^	1.19 ^d^	0.037	0.080	<0.001	<0.001
Nitrogen use efficiency ^6^ (g/g)	0.174	0.166	0.0039	0.204 ^a^	0.170 ^bc^	0.183 ^b^	0.158 ^cd^	0.165 ^c^	0.166 ^c^	0.146 ^d^	0.0051	0.162	<0.001	<0.001

^1^ SEM, standard error of mean; ^2 a–d^ different letters on the shoulder of values in a row under the sample time columns mean a significant difference (*p* < 0.05); ^3^ ECM, energy-corrected milk and was calculated as: ECM (kg) = 0.3246 × milk yield (kg) + 13.86 × milk fat yield (kg) + 7.04 × milk protein yield (kg) [27]; ^4^ SNF, milk solids not fat; ^5^ DMI, dry matter intake; ^6^ Nitrogen use efficiency was calculated as milk nitrogen output/feed nitrogen input.

**Table 3:** Plasma biochemical parameters of dairy cows fed experimental diets supplemented without (CON) or with (LPL) lysophospholipids.

Index	Treatment		SEM ^1^	Sampling Time				SEM	*p* Value		
	CON	LPL		Day 0	Day 14	Day 28	Day 42		Treat	Time	Treat × Time
Total protein (TP) (g/L)	70.16 ^b 2^	77.78 ^a^	1.886	76.40 ^a^	69.01 ^b^	73.04 ^ab^	77.42 ^a^	2.543	0.017	0.097	0.267
Albumin (ALB) (g/L)	30.14	29.87	0.844	30.09	28.50	30.43	30.99	0.893	0.828	0.144	0.474
Globulin (GLOB) (g/L)	40.02 ^b^	47.90 ^a^	1.796	46.31 ^a^	40.51 ^b^	42.61 ^b^	46.42 ^a^	2.540	0.004	0.285	0.225
Albumin/ Globulin	0.787 ^a^	0.641 ^b^	0.0320	0.697	0.726	0.742	0.691	0.0452	0.003	0.840	0.255
Triglyceride (TG) (mmol/L)	0.161	0.170	0.0061	0.174	0.163	0.170	0.153	0.0086	0.316	0.356	0.655
Total cholesterol (TC) (mmol/L)	5.46 ^a^	4.65 ^b^	0.251	5.76	4.62	4.87	4.98	0.354	0.027	0.138	0.917
Urea nitrogen (PUN) (mmol/L)	5.51	5.77	0.226	4.85 ^c^	5.78 ^b^	5.46 ^b^	6.46 ^a^	0.320	0.390	<0.001	0.436
Glucose (GLU) (mmol/L)	3.25	3.38	0.078	3.19 ^bc^	3.11 ^c^	3.34 ^b^	3.63 ^a^	0.084	0.252	<0.001	0.231
Alkaline phosphatase (ALP) (IU/L)	30.23 ^b^	42.12 ^a^	2.127	33.17	37.00	36.85	37.70	3.008	<0.001	0.686	0.276

^1^ SEM, standard error of mean; ^2 a–d^ different letters on the shoulder of values in a row under the treatment columns or sample time columns mean a significant difference (*p* < 0.05).
